# Screen Time and Parents’ Education Level Are Associated with Poor Adherence to the Mediterranean Diet in Spanish Children and Adolescents: The PASOS Study

**DOI:** 10.3390/jcm10040795

**Published:** 2021-02-16

**Authors:** Julia Wärnberg, Napoleón Pérez-Farinós, Juan Carlos Benavente-Marín, Santiago Felipe Gómez, Idoia Labayen, Augusto G. Zapico, Narcis Gusi, Susana Aznar, Pedro Emilio Alcaraz, Miguel González-Valeiro, Lluís Serra-Majem, Nicolás Terrados, Josep A. Tur, Marta Segú, Camille Lassale, Clara Homs, Maddi Oses, Marcela González-Gross, Jesús Sánchez-Gómez, Fabio Jiménez-Zazo, Elena Marín-Cascales, Marta Sevilla-Sánchez, Estefanía Herrera-Ramos, Susana Pulgar, María del Mar Bibiloni, Olga Sancho-Moron, Helmut Schröder, F. Javier Barón-López

**Affiliations:** 1 Epi-Phaan Research Group, School of Health Sciences, Universidad de Málaga, Instituto de Investigación Biomédica de Málaga (IBIMA), 29071 Málaga, Spain; jwarnberg@uma.es (J.W.); jc.benaventemarin@gmail.com (J.C.B.-M.); baron@uma.es (F.J.B.-L.); 2 Centro de Investigación Biomédica en Red Fisiopatología de la Obesidad y la Nutrición (CIBEROBN), Institute of Health Carlos III, 28029 Madrid, Spain; lluis.serra@ulpgc.es (L.S.-M.); pep.tur@uib.es (J.A.T.); classale@imim.es (C.L.); marcela.gonzalez.gross@upm.es (M.G.-G.); mar.bibiloni@uib.es (M.d.M.B.); 3 Epi-Phaan Research Group, School of Medicine, Universidad de Málaga, Instituto de Investigación Biomédica de Málaga (IBIMA), 29071 Málaga, Spain; 4 Programs, Gasol Foundation, Sant Boi de Llobregat, 08830 Barcelona, Spain; sgomez@gasolfoundation.org (S.F.G.); choms@gasolfoundation.org (C.H.); 5 GREpS, Health Education Research Group, Nursing and Physiotherapy Department, University of Lleida, 25198 Lleida, Spain; 6 ELIKOS Group, Institute for Innovation and Sustainable Development in Food Chain (IS-FOOD), Instituto de Investigación Sanitaria de Navarra, Public University of Navarre, 31006 Pamplona, Spain; idoia.labayen@unavarra.es (I.L.); maddi.oses@unavarra.es (M.O.); 7 ImFINE Research Group, Department of Health and Human Performance, Universidad Politecnica de Madrid, 28040 Madrid, Spain; azapico@edu.ucm.es; 8 Department of Didactics of Language, Arts and Physical Education, Universidad Complutense de Madrid, 28040 Madrid, Spain; 9 Physical Activity and Quality of Life Research Group (AFYCAV), Faculty of Sport Sciences, University of Extremadura, 10003 Caceres, Spain; narcis.gusi@gmail.com (N.G.); jesanchezg@unex.es (J.S.-G.); 10 PAFS Research Group, Faculty of Sports Sciences, University of Castilla-La Mancha-Toledo Campus, 45071 Toledo, Spain; Susana.Aznar@uclm.es (S.A.); Fabio.jimenez@uclm.es (F.J.-Z.); 11 Research Center for High Performance Sport, San Antonio Catholic University of Murcia, 30830 Murcia, Spain; palcaraz@ucam.edu (P.E.A.); emarin@ucam.edu (E.M.-C.); 12 Faculty of Sport Sciences, San Antonio Catholic University of Murcia, 30107 Murcia, Spain; 13 Faculty of Sports Sciences and Physical Education, Universidade da Coruña, 15179 A Coruña, Spain; miguel.gonzalez.valeiro@udc.es (M.G.-V.); marta.sevilla@udc.es (M.S.-S.); 14 Research Institute of Biomedical and Health Sciences (IUIBS), University of Las Palmas de Gran Canaria, 35016 Las Palmas, Spain; faniya1@gmail.com; 15 Regional Unit of Sports Medicine–Municipal Sports Foundation of Avilés and Health Research Institute of the Principality of Asturias (ISPA), 33401 Avilés, Spain; nterrados@aviles.es (N.T.); susanapulgarmunoz@gmail.com (S.P.); 16 Research Group of Community Nutrition and Oxidative Stress, University of the Balearic Islands and IDISBA, 07122 Palma de Mallorca, Spain; 17 Probitas Foundation, 08022 Barcelona, Spain; marta.segu@fundacionprobitas.org (M.S.); olga.sancho@grifols.com (O.S.-M.); 18 Cardiovascular Risk and Nutrition Research Group, Hospital del Mar Institute for Medical Research (IMIM), 08003 Barcelona, Spain; HSchoeder@imim.es; 19 Global Research on Wellbeing (GRoW), Blanquerna Ramon Llull University Faculty of Health Sciences, 08025 Barcelona, Spain; 20 Centro de Investigación Biomédica en Red Fisiopatología de Epidemiología y Salud Pública (CIBERESP), Institute of Health Carlos III, 28029 Madrid, Spain

**Keywords:** screen time, education level, eating pattern, Mediterranean diet

## Abstract

The aim of this study is to evaluate if screen time and parents’ education levels are associated with adherence to a Mediterranean dietary pattern. This cross-sectional study analyzed a representative sample of 3333 children and adolescents (8 to 16 years) included in the Physical Activity, Sedentarism, lifestyles and Obesity in Spanish youth (PASOS) study in Spain (which ran from March 2019 to February 2020). Data on screen time (television, computer, video games, and mobile phone) per day, Mediterranean diet adherence, daily moderate or vigorous physical activity, and parents’ education levels were gathered using questionnaires. A descriptive study of the variables according to sex and parents’ education level was performed. Logistic regression models (adjusted by sex and weight status) were fitted to evaluate the independent association between screen time and Kids’ level of adherence to the Mediterranean diet (KIDMED) index, as well as some of its items. A greater amount of screen time was associated with worse adherence to the Mediterranean diet; a lower consumption of fruit, vegetables, fish, legumes, and nuts; and a greater consumption of fast food, sweets, and candies. A lower parents’ education level was associated with worse adherence to the Mediterranean diet. It is necessary to promote the responsible, limited use of screen time, especially in children with parents with a lower education level.

## 1. Introduction

Childhood obesity is a significant public health problem worldwide [1,2]. In addition to its short-term health consequences, it is related to obesity in adulthood and thus to numerous other diseases that cause a high level of mortality, morbidity, and disability [3,4]. Obesity is highly prevalent in children and adolescents, especially in certain countries or regions, including southern Europe and Spain [5,6,7]. Childhood obesity is related to an imbalance between energy intake and expenditure, which leads to an increase in weight. This increase in weight has been associated with different lifestyle factors, such as diet, sedentary behavior, and physical activity [8,9,10]. Among dietary patterns, the Mediterranean diet is defined as an eating pattern that includes the intake of a large amount of fruit, vegetables, legumes, grains, nuts, and olive oil; moderate consumption of fish, dairy products, and eggs; and reduced consumption of meat. The Mediterranean diet has been demonstrated to be a dietary pattern that has favorable effects on health, not just in terms of weight control, but also in the prevention of non-communicable diseases [11,12]. Therefore, it is an ideal dietary pattern during childhood and adolescence. Ceasing to follow this dietary pattern could lead to health deficiencies in children and adolescents [13] and, as such, it is important to know the level of adherence to the Mediterranean diet and factors that influence it.

In addition to diet, other factors have been studied as determinants of childhood obesity. Among them are those related to physical activity and sedentarism. Various studies, such as those by Bawaked et al. and Arriscado et al., have documented that low levels of physical activity are associated with a lower degree of adherence to the Mediterranean diet among children between 8 and 12 years of age [14,15]. In addition to low levels of activity, a greater amount of time spent on sedentary activities seems to be related to lower adherence to the Mediterranean diet. Among these sedentary activities, screen time is being studied as a factor associated with unhealthy dietary patterns. The mechanisms by which screen time is associated with childhood and adolescent obesity are not entirely clear. Apart from the substitution of physical activity with sedentary entertainment [16,17], it has been suggested that increased energy intake may be associated with watching television or playing video games through a reduction in the sensation of satiety and the fostering of unhealthy eating habits [18]. Other factors have also been considered, such as children’s exposure to advertising for food and beverages that are high in saturated fats, sugars, or salt, which could promote their desire to consume these products. Likewise, screen time is interrelated with insufficient sleep time in children which, through endocrine mechanisms, could favor childhood and adolescent obesity by means of its effects on eating patterns [19,20]. Indeed, the amount of time per day that children and adolescents spend on passive screen time entertainment such as television, mobile telephones, computers, and video games, as well as having a television in their room, seem to play a role [21,22,23]. A recent study by Tapia Serrano et al. found that a greater amount of screen time was associated with worse adherence to the Mediterranean diet in adolescents (12 to 14 years of age) in Extremadura (western Spain) [13].

In addition to the aforementioned factors, parents’ education level also appears to be associated with factors that may influence childhood and adolescent obesity, such as dietary and physical activity habits [24,25]. For years, education level has been considered to be one of the strongest predictors of health and mortality, even more so than income level [26]. Therefore, we believe it is relevant to evaluate parents’ education level as a determining factor in dietary habits and, in this case, in adherence to the Mediterranean diet in children and adolescents.

Studies have been conducted in Spain which evaluate the association between screen time and Mediterranean diet and dietary patterns, but the sample sizes have been small, the age groups have been narrow, or they have only focused on certain regions [13,14,15,27,28]. A study such as this one, with a large sample size that is representative of Spain’s child and adolescent population, is necessary.

The aims of this study are first to evaluate if screen time is associated with the Mediterranean dietary pattern in children and adolescents after adjusting for other variables, and second to determine adherence to the Mediterranean diet according to parents’ education level.

## 2. Materials and Methods

### 2.1. Design, Setting, and Participants

This work is a cross-sectional study based on the Physical Activity, Sedentarism, lifestyles and Obesity in Spanish youth (PASOS) study. A complete description of the design and methodology of the PASOS study has previously been published [29]. The PASOS study is a multicenter, cross-sectional, population-based study conducted in a representative sample of the Spanish population aged 8 to 16 years. Its objective is to determine levels of physical activity, sedentarism, lifestyle factors, and weight status.

Randomization of the participating schools was performed by means of a multistage sampling procedure, as previously described. Data were collected between March 2019 and February 2020 in 244 primary and secondary schools across all 17 Spanish regions. Informed consent was obtained from 4092 parents or guardians some weeks before the data collection day. A parental questionnaire was also sent home in paper format to be self-reported and sent back to school. On one occasion during school hours, trained researchers visited the participating school for data collection of children and adolescents. Anthropometric measurements of children and adolescents were taken by trained personnel. Questionnaires were self-reported by the children and adolescents online at school in the presence of trained personnel who were available to answer any possible questions.

From 4092 children and adolescents whose parents signed the consent, 326 were excluded because they were under 8 or over 16 years old, 216 were excluded because they did not attend school the day the data collection took place, and 217 children and adolescents were excluded because their parents or guardians did not return the questionnaire with information about their level of education. This analysis included 3333 children and adolescents aged between 8 and 16.99 years who had complete information on all variables of interest. These included being present at school on the day data were collected and having anthropometric measurements taken; having valid data from the Kids’ level of adherence to the Mediterranean diet (KIDMED), screen time use, and Physical Activity Physical Activity Unit 7 item Screener (PAU-7S) questionnaires; and having the parental questionnaire responded to by at least one parent or guardian.

### 2.2. Study Variables

#### 2.2.1. Outcome Variables

The outcome variables were those related to the adherence to the Mediterranean diet of children and adolescents. The KIDMED index evaluates the habitual diet and degree of adherence to the Mediterranean diet. It was developed and has been validated in Spanish children and adolescents [12,30,31].

The questionnaire includes 16 yes-or-no questions: 4 questions that denote low adherence and have a score of −1, and 12 questions that denote high adherence and have a score of +1. Therefore, the KIDMED index score ranges from −4 to +12 points. The KIDMED index was also used to classify participants into three categories: low adherence (3 or fewer points), medium adherence (4–7 points), and high adherence (8 or more points) [31]. For multivariate models (binary logistic regression), the KIDMED index was coded into two categories: high adherence and medium or low adherence.

Twelve of the 16 questionnaire items corresponding to frequency of intake of specific food groups were evaluated and are presented individually: one serving of fruit per day, more than one serving of fruit per day, one serving of fresh or cooked vegetables per day, more than one serving of fresh or cooked vegetables per day, at least 2–3 servings of fish per week, at least one serving of legumes per week, at least 5 servings of pasta or rice per week, at least one serving of dairy products (yogurt, milk) per day, at least 2–3 servings of nuts or seeds per week, the use of olive oil at home, sweets or candy several times per day and eating at a fast food restaurant one or more times per week.

#### 2.2.2. Exposure Variable

The exposure variables were mean daily screen time measured in hours and parents’ education level. In the PASOS study, screen time was evaluated using the Healthy Lifestyle in Europe by Nutrition in Adolescence (HELENA) (study’s screen time-based sedentary behavior questionnaire [32], which asks about the time spent per day on four activities: watching television, playing on the computer, playing video games, and using a mobile telephone. It enquires about weekdays and weekends separately. To calculate mean daily time, the weighted mean was calculated for weekdays (5 days) and weekends (2 days).

Parents’ education level was classified into three categories: “no formal or only primary school education”, “secondary school”, or “university”. When the parents had different education levels, the highest one was chosen. Children and adolescents were then classified into one of three categories according to the parents’ education level.

#### 2.2.3. Covariates

The sex and age of children and adolescents were reported by parents on the informed consent form. In addition, the following covariates were selected from among those that were collected in the PASOS study: participants’ weight status and mean daily and weekly time spent doing moderate or vigorous physical activity (MVPA). These covariates were chosen because as they are related to the dependent variable and exposure variables, it is believed they may have a confounding effect on the estimations [10,13,33].

Children and adolescents’ weight status was assessed using body mass index (BMI), calculated using the formula: weight (kg)/height^2^ (m^2^). Weight and height were measured according to the World Health Organization (WHO) standardized protocol [34]. Body weight was measured to the nearest 100 g using an electronic scale (SECA 899, SECA GmbH, Hamburg, Germany), and height was measured to the nearest 1 mm with a portable stadiometer (SECA 217, SECA GmbH, Hamburg, Germany). The weight status variable was classified into two categories: normal weight and excess weight, which included overweight and obesity. The BMI-for-age cut-off points of the WHO child growth standards were used to define overweight and obesity [35].

The self-administered PAU-7S questionnaire, which consists of seven questions on physical activity, was used to estimate mean time spent daily (in minutes) and weekly (in hours) doing MVPA [29]. The PAU-7S questionnaire has been validated in a randomized sample of 321 participants from the PASOS study that completed the PAU-7S twice and wore an accelerometer (reference method) for at least 7 consecutive days. It showed a good internal consistency (Cronbach’s alpha: 0.76), a fair correlation with data measured with an accelerometer (Spearman’s correlation coefficient: 0.62), and a fair concordance (kappa value: 0.24) (unpublished data).

### 2.3. Statistical Analysis

A descriptive study of the variables was conducted, calculating the mean and standard deviation for quantitative variables (age, daily screen time, MVPA/week, MVPA/day, and KIDMED index score) and absolute frequency and percentage for qualitative variables (age group, sex, categories of KIDMED index, and KIDMED questionnaire items). This descriptive study was conducted on the total sample according to parents’ education level group and according to participants’ sex. Differences among the groups were evaluated using the chi-square test for qualitative variables (with Bonferroni-corrected *p*-values for pairwise comparisons) and the ANOVA test for quantitative variables (with Bonferroni corrections for multiple testing). Likewise, a descriptive analysis of the quantitative variables was performed separately for children and adolescents.

To assess the independent association between screen time and adherence to the Mediterranean diet, various multivariate models of binary logistic regressions were adjusted. In the first, the dependent variable was adherence to the Mediterranean diet as measured by the KIDMED index (high adherence and medium or low adherence) and the independent variables were mean daily screen time (hours) and parents’ education level. The model was adjusted by sex and weight status and was conducted separately in children and adolescents. In addition, other models were fitted, one for each KIDMED index item as the dependent variable and screen time as the independent variable, adjusted for sex, parents’ education level, weight status, and MVPA.

### 2.4. Ethical Aspects

The PASOS study was approved by the Ethics Committee of the Fundació Sant Joan de Déu, Barcelona, Spain. A signed informed consent form was obtained from the parents or legal guardians of each participant. The study was performed according to the guidelines of the Declaration of Helsinki.

## 3. Results

A description of the total sample and of participants according to parents’ education level can be seen in Table 1 and Table 2. A total of 1500 participants (45.0%) were between 8 and 11 years of age (children,) and 1833 (55.0%) were between 12 and 16 years of age (adolescents). Of the total sample, 52.1% were girls and 47.9% were boys. No differences were observed in parents’ education level according to participants’ sex.

Mediterranean diet adherence was low in 10.2%, medium in 49.3%, and high in 40.5% of the total population. Adherence was high in 49.6% of children whose parents had a university education, in 36.3% of children whose parents had a secondary school education, and in 31.3% of children whose parents had a primary school or no formal education. These differences were statistically significant. A significantly higher percentage of children whose parents had a university education consumed fruit, vegetables, and dairy products daily, and fish, legumes, and nuts weekly. A significantly higher percentage of children whose parents had secondary or primary/no formal education consumed more fast food and sweets and candy (Table 1).

The mean KIDMED index score was significantly higher in children and adolescents whose parents had a university education than children whose parents had either secondary or primary/no formal education (Table 2).

Mean daily screen time was 206.5 min in the total sample (Table 2). Among children aged 8 to 11 years, mean daily screen time was significantly lower in children whose parents had a university education level than in children whose parents had a secondary or primary/no formal education. Among adolescents, mean daily screen time was also significantly lower in children whose parents had a university education level than in those whose parents had a secondary or primary/no formal education.

MVPA time per day was also significantly higher in children and adolescents whose parents had a university education level with respect to those whose parents had secondary or primary/no formal education (Table 2). There were no differences between children and adolescents whose parents had secondary education and those whose parents had primary/no formal education.

There were no differences according to sex on the KIDMED index, but there were differences in screen time and physical activity (Table 3). In both children and adolescents, mean daily screen time was significantly higher among boys than girls. MVPA time per day was also significantly higher for boys than girls, both among children and adolescents.

The adjusted odds ratio (OR) and 95% confidence intervals (CI) of the multivariate analysis can be seen in Table 4 and Table 5. An additional hour of screen time was significantly associated with a greater probability of medium or low adherence to the Mediterranean diet, as measured by the KIDMED index, both in children (OR: 1.18; 95% CI: 1.11–1.24) and adolescents (OR: 1.23; 95% CI: 1.17–1.27). Low education levels were also significantly associated with worse adherence to the Mediterranean diet in children and adolescents (Table 4). On the other hand, one additional hour of screen time in children was significantly associated with a lower probability of daily fruit and vegetable consumption, daily consumption of more than one serving of fruit, and legume consumption more than once per week and with a greater probability of fast food consumption one or more times per week and the consumption of sweets or candy several times per day. Among adolescents, similar statistically significant associations were also found, except for the daily consumption of more than one serving of vegetables (Table 5).

## 4. Discussion

Numerous studies have examined the relationship between time spent on passive entertainment such as television, mobile telephones, or video games and the presence of or predisposition to childhood and adolescent obesity [36,37,38,39,40,41,42,43,44,45]. However, few studies have been conducted on screen time and its association with eating patterns [46,47,48]. This study found evidence that in children and adolescents of both sexes, an increase in screen time is associated with a low adherence to the Mediterranean diet; greater consumption of fast food, sweets, and candy; and lower consumption of fruit, vegetables, legumes, dairy products, fish, and nuts. This association is consistent with what is reported in the scientific literature [37,49,50]. Estimated screen time is greater among adolescents than children, which is also in line with what has been noted in other studies [25,51].

One possible explanation for these results could be that consumption of products rich in saturated fats, salt, or sugar is higher among children and adolescents who spend more time in front of screens because they pay less attention to what they eat while they are doing another activity [40,46]. In addition, many of the advertisements on television or the internet, especially during programs aimed at children or young people, are for food and beverages that have high energy, salt, fat, or sugar content [52,53,54].

In this study, a consistent association has been observed between screen time and fruit and vegetable consumption such that the greater the amount of screen time, the lower the probability of daily fruit or vegetable consumption or daily consumption of more than one serving of fruit. These results are highly relevant because very few studies have documented this association. López-Gil et al. observed a similar relationship for children, but their findings were only among girls, in a non-representative and smaller sample size than ours, and in a Spanish region that produces a lot of fruit and vegetables [55].

The decrease in daily fruit and vegetable consumption associated with a greater amount of screen time may be related to a substitution effect; fruit and vegetables may be in competition with different groups of products with a high energy content, which encompasses a very wide range of products [56]. This occurs despite the fact that high exposure to advertisements and campaigns for fruit and vegetables is associated with a greater consumption of these products [57].

Our study also noted an association between screen time and a greater consumption of food in fast food establishments, as well as of sweets and candy. Similar observations were noted in recent studies by Benaich et al. [58] and Vizcaino et al. [59].

Among the results obtained, it was found that screen time is significantly higher among boys than girls, both in children and adolescents. These differences were also reported in a recent study by Myszkowska-Ryciak et al. [60]. This finding must be taken into consideration when it comes to planning targeted and general prevention strategies.

It is important to note that the results of our research show an association between screen time and a low adherence to the Mediterranean diet that differ according to parents’ education level such that the lower the education level, the greater the screen time. A work by Abdel Magid et al. showed that among adolescents in the United States of America, screen time was higher in those whose parents had a low level of education [61]. Our results are also in line with a study by Pons et al. which indicated that a high education level among mothers is associated with lesser screen time and other healthy habits [14,25]. The mechanism which underlies the association between a lower educational level and a greater amount of screen time is not entirely clear. However, some theoretical models have been proposed. Various studies maintain that this association can be explained by the impact of education on healthy lifestyles, more so than better access to resources [62,63]. The SLOTH model, developed by Cawley in 2004, considers the allocation of time to various activities. This model assumes that individuals choose how to spend their time according to utility and cost [64,65]. Thus, the decision to spend time on passive entertainment activities is related to a lower level of education and this could be reflected in children’s behavior.

Especially noteworthy are the results regarding Mediterranean diet adherence, as measured by the KIDMED index, according to parents’ education level. They show that nearly half of children and adolescents whose parents had a university education had high adherence to the Mediterranean diet, whereas the percentage was much lower among children whose parents had a low or medium education level. Outcomes that are consistent with ours in this respect have been observed in studies conducted in various countries [66,67,68,69]. Yañez et al. have shown that low family educational level is associated with low adherence to the Mediterranean diet in adolescents [70]. It has been described that a low parent education level is associated with little knowledge of nutrition and a low level of awareness about aspects related to nutrition. In these cases, consumers can make mistakes when following nutritional recommendations and healthy diet guidelines [71]. This may explain the low adherence to the Mediterranean diet among children with parents with a lower education level. This leads one to think that in addition to general actions aimed at raising awareness and building adequate food advertising regulations, strategies and interventions aimed at more disadvantaged social classes must also be proposed.

The strengths of this study are that it approaches a topic that has not often been directly studied: the association between time spent on passive entertainment and eating patterns among children and adolescents. Second, it was conducted within the PASOS study on a large, representative sample of the child and adolescent population in Spain and among a wide age range, which provides consistent estimates. Third, the methodology used is rigorous and standardized among all sections, and this study uses validated tools administered by specifically trained personnel. The main limitation of this study is its cross-sectional nature, which makes it impossible to determine the relationship between cause and effect in the associations found. Another limitation could be related to the fact that the information used for screen time and dietary patterns is self-reported, which could have led to less precise estimates. Our study did not examine the influence of parents’ education level according to their sex, but this is another line of research that could shed light on new data of interest.

## 5. Conclusions

Screen time is associated with a low adherence to the Mediterranean diet in both children (8–11 years) and adolescents (12–16 years). It is also associated with a lower consumption of fruit, vegetables, and legumes, as well as a greater consumption of fast food, sweets, and candy. Screen time is higher among boys than girls in both children and adolescents whose parents have a low education level.

Adopting healthy eating habits goes hand-in-hand with improvements in other lifestyle aspects, such as physical activity and sedentarism. Among children and adolescents, passive screen time entertainment must be used in a limited, responsible manner in order to avoid excess use. Education is one of the keys to improving dietary and physical activity habits. In addition to general strategies to improve lifestyles and generate healthy environments, specific strategies aimed at social classes with lower education levels must be designed.

## Figures and Tables

**Table 1:** Characteristics of study participants: age, sex, and adherence to the Mediterranean diet for the total study population and according to parents’ education level. KIDMED: Kids’ level of adherence to the Mediterranean diet.

Variables		Total		Primary School or No School		Secondary School		University		
		*n*	%	*n*	%	*n*	%	*n*	%	*p*
Age										
	Children, 8–11 years	1500	45.0	165	11.0	764	50.9	571	38.1	
	Adolescents, 12–16 years	1833	55.0	203	11.1	1014	55.3	616	33.6	
Sex										
	Boys	1595	47.9	189	11.8	833	52.2	573	35.9	
	Girls	1738	52.1	179	10.3	945	54.4	614	35.3	0.271
Mediterranean diet adherence (KIDMED index)										
	Low	339	10.2	42	11.4 ^a^	226	12.7 ^a^	71	6.0 ^b^	
	Medium	1644	49.3	211	57.3 ^a^	906	51.0 ^b^	527	44.4 ^c^	
	High	1350	40.5	115	31.3 ^a^	646	36.3 ^a^	589	49.6 ^b^	<0.001
Daily fruit consumption										
	No	926	27.8	103	28.0 ^a^	548	30.8 ^a^	275	23.2 ^b^	
	Yes	2407	72.2	265	72.0 ^a^	1230	69.2 ^a^	912	76.8 ^b^	<0.001
Daily consumption of more than one serving of fruit										
	No	1640	49.2	181	49.2 ^a^	963	54.2 ^a^	496	41.8 ^b^	
	Yes	1693	50.8	187	50.8 ^a^	815	45.8 ^a^	691	58.2 ^b^	<0.001
Daily vegetable consumption										
	No	1198	35.9	148	40.2 ^a^	684	38.5 ^a^	366	30.8 ^b^	
	Yes	2135	64.1	220	59.8 ^a^	1094	61.5 ^a^	821	69.2 ^b^	<0.001
Daily consumption of more than one serving of vegetables										
	No	2260	67.8	253	68.8 ^a^	1229	69.1 ^a^	778	65.5 ^a^	
	Yes	1073	32.2	115	31.3 ^a^	549	30.9 ^a^	409	34.5 ^a^	0.114
Fish consumption at least 2–3 times per week										
	No	1260	37.8	152	41.3 ^a^	741	41.7 ^a^	367	30.9 ^b^	
	Yes	2073	62.2	216	58.7 ^a^	1037	58.3 ^a^	820	69.1 ^b^	<0.001
Legume consumption at least once per week										
	No	1018	30.5	129	35.1 ^a^	561	31.6 ^a,b^	328	27.6 ^b^	
	Yes	2315	69.5	239	64.9 ^a^	1217	68.4 ^a,b^	859	72.4 ^b^	0.010
Pasta or rice consumption 5 or more times per week										
	No	1718	51.5	185	50.3 ^a^	887	49.9 ^a^	646	54.4 ^b^	
	Yes	1615	48.5	183	49.7 ^a^	891	50.1 ^a^	541	45.6 ^b^	0.047
Consumption of at least one serving of dairy products per day										
	No	741	22.2	98	26.6 ^a^	411	23.1 ^a,b^	232	19.5 ^b^	
	Yes	2592	77.8	270	73.4 ^a^	1367	76.9 ^a,b^	955	80.5 ^b^	0.007
Nuts consumption at least 2–3 times per week										
	No	1604	48.1	189	51.4 ^a^	893	50.2 ^a^	522	44.0 ^b^	
	Yes	1729	51.9	179	48.6 ^a^	885	49.8 ^a^	665	56.0 ^b^	0.002
Use of olive oil at home										
	No	287	8.6	38	10.3 ^a^	163	9.2 ^a^	86	7.2 ^a^	
	Yes	3046	91.4	330	89.7 ^a^	1615	90.8 ^a^	1101	92.8 ^a^	0.087
Sweets and candy consumption several times per day										
	No	2616	78.5	270	73.4 ^a^	1340	75.4 ^a^	1006	84.8 ^b^	
	Yes	717	21.5	98	26.6 ^a^	438	24.6 ^a^	181	15.2 ^b^	<0.001
Consumption of fast food at least once per week										
	No	2572	77.2	263	71.5 ^a^	1324	74.5 ^a^	985	83.0 ^b^	
	Yes	761	22.8	105	28.5 ^a^	454	25.5 ^a^	202	17.0 ^b^	<0.001

*p* value for the chi-square test among education levels and the variables studied. ^a,b,c^ different letters mean *p* < 0.05 on the comparisons between education level categories (Bonferroni-corrected *p*-values).

**Table 2:** Characteristics of study participants: age, screen time, physical activity, and KIDMED index for the total study population and according to parents’ education level.

	Total		Children (8–11 Years)							Adolescents (12–16 Years)						
			Primary School or No School		Secondary School		University			Primary School or No School		Secondary School		University		
Variables	Mean	SD	Mean	SD	Mean	SD	Mean	SD		Mean	SD	Mean	SD	Mean	SD	
Age (years)	12.5	2.3	10.3 ^a^	1.0	10.4 ^a^	1.0	10.4 ^a^	1.0		14.3 ^a^	1.3	14.4 ^a^	1.4	14.2 ^a^	1.3	
Daily screen time (minutes)	207.9	143.2	173.3 ^a^	131.9	170.9 ^a^	139.9	125.0 ^b^	112.2	*	266.2 ^a^	143.7	267.7 ^a^	139.2	222.2 ^b^	127.3	*
MVPA/week (minutes)	870.0	540.3	914 ^a^	498.5	938.7 ^a^	512.9	1004.0 ^b^	512.2	*	764.2 ^a^	531.5	765.3 ^a^	547.8	855.7 ^b^	560.0	*
MVPA/day (minutes)	124.3	77.2	130.6 ^a^	71.2	134.1 ^a^	73.3	143.4 ^b^	73.2	*	109.2 ^a^	75.9	109.3 ^a^	78.3	122.2 ^b^	80.0	*
KIDMED index	6.8	2.5	6.7 ^a^	2.5	6.8 ^a^	2.5	7.6 ^b^	2.4	*	6.1 ^a^	2.3	6.2 ^a^	2.5	7.2 ^b^	2.3	*

SD: standard deviation; MVPA: moderate or vigorous physical activity. *, *p* < 0.05 for the ANOVA between education level categories. ^a,b^ different letters mean *p* < 0.05 on the comparison between education level categories (Bonferroni correction for multiple tests).

**Table 3:** Characteristics of study participants: age, screen time, physical activity, and KIDMED index for the total study population and according to sex.

	Total		Children (8–11 Years)					Adolescents (12–16 Years)				
			Boys		Girls			Boys		Girls		
Variables	Mean	SD	Mean	SD	Mean	SD		Mean	SD	Mean	SD	
Age (years)	12.5	2.3	10.4	1.0	10.4	1.0		14.3	1.3	14.3	1.3	
Daily screen time (minutes)	207.9	143.2	184.0	144.8	124.5	108.6	*	271.6	144.7	235.1	128.3	*
MVPA/week (minutes)	870.0	540.3	1037.9	518.3	886.6	494.7	*	929.9	554.9	677.1	520.8	*
MVPA/day (minutes)	124.3	77.2	148.3	74.0	126.7	70.7	*	132.8	79.3	96.7	74.4	*
KIDMED index	6.8	2.5	7.0	2.5	7.1	2.5		6.7	2.4	6.4	2.5	*

*, *p* < 0.05 for the difference of means between sexes.

**Table 4:** Adjusted odds ratio (OR) and 95% confidence interval (CI) for the effects of screen time and parents’ education level on low or medium Mediterranean diet adherence (KIDMED index < 8).

		Children (8–11 Years)				Adolescents (12–16 Years)			
Variables		OR	95% CI		*p*	OR	95% CI		*p*
Daily screen time (hours)		1.178	1.116	1.243	<0.001	1.225	1.167	1.286	<0.001
Education level									
	University	1				1			
	Secondary school	1.378	1.102	1.724	0.005	1.641	1.327	2.029	<0.001
	Primary school or no school	1.117	0.902	1.383	0.003	2.192	1.531	3.138	<0.001

Adjusted for sex and weight status.

**Table 5:** Adjusted OR and 95% CI for the effects of screen time on dietary habits selected from the KIDMED index.

	Children (8–11 Years)				Adolescents (12–16 Years)			
Variables	OR	95% CI		*p*	OR	95% CI		*p*
Daily fruit consumption	0.925	0.875	0.978	0.006	0.895	0.856	0.936	<0.001
Daily consumption of more than one serving of fruit	0.886	0.843	0.933	<0.001	0.901	0.862	0.942	<0.001
Daily vegetable consumption	0.915	0.870	0.962	<0.001	0.914	0.876	0.955	<0.001
Daily consumption of more than one serving of vegetables	0.929	0.880	0.980	0.007	0.988	0.943	1.035	0.609
Fish consumption at least 2–3 times per week	0.903	0.858	0.949	<0.001	0.915	0.877	0.955	<0.001
Legume consumption more than once per week	0.898	0.853	0.944	<0.001	0.920	0.880	0.962	<0.001
Pasta or rice consumption 5 or more times per week	1.055	1.004	1.108	0.032	1.054	1.011	1.099	0.013
Consumption of at least one serving of dairy products per day	1.025	0.969	1.084	0.397	1.014	0.962	1.068	0.614
Nuts consumption at least 2–3 times per week	0.941	0.896	0.989	0.016	0.966	0.926	1.007	0.106
Use of olive oil at home	1.003	0.934	1.077	0.936	1.057	0.961	1.164	0.255
Sweets and candy consumption several times per day	1.206	1.140	1.276	<0.001	1.256	1.196	1.319	<0.001
Consumption of fast food at least once per week	1.168	1.106	1.234	<0.001	1.192	1.137	1.249	<0.001

Adjusted for sex, parents’ education level, weight status, and MVPA.

## Data Availability

Data sharing not applicable.
